# Identification of immune-related molecular markers in intracranial aneurysm (IA) based on machine learning and cytoscape-cytohubba plug-in

**DOI:** 10.1186/s12863-023-01121-w

**Published:** 2023-04-11

**Authors:** Zhengfei Ma, Ping Zhong, Peidong Yue, Zhongwu Sun

**Affiliations:** 1 Department of Neurology, The First Affiliated Hospital of Anhui Medical University, Anhui Province, No. 299, Bianhe Zhong Lu District, Suzhou City, Hefei, 234000 China; 2 Department of Neurology, Suzhou Hospital of Anhui Medical University, Suzhou, China; 3 Department of Neurosurgery, Suzhou Hospital of Anhui Medical University, Suzhou, China

**Keywords:** Intracranial aneurysm, Immune cell infiltration, Machine learning, Diagnose, Differentially expressed mRNAs

## Abstract

**Background:**

Intracranial aneurysm (IA) is a common cerebrovascular disease. The immune mechanism of IA is more complicated, and it is unclear so far. Therefore, it is necessary to continue to explore the immune related molecular mechanism of IA.

**Methods:**

All data were downloaded from the public database. Limma package and ssGSEA algorithm was used to identify differentially expressed mRNAs (DEmRNAs) and analyze immune cell infiltration, respectively. Machine learning and cytoscape-cytohubba plug-in was used to identify key immune types and multicentric DEmRNAs of IA, respectively. Multicentric DEmRNAs related to key immune cells were screened out as key DEmRNAs by Spearman correlation analysis. Diagnostic models, competing endogenous RNA (ceRNA) regulatory network and transcription factor regulatory network were constructed based on key DEmRNAs. Meanwhile, drugs related to key DEmRNAs were screened out based on DGIdb database. The expression of key DEmRNAs was also verified by real time-PCR.

**Results:**

In this study, 7 key DEmRNAs (NRXN1, GRIA2, SLC1A2, SLC17A7, IL6, VEGFA and SYP) associated with key differential immune cell infiltration (CD56bright natural killer cell, Immature B cell and Type 1 T helper cell) were identified. Functional enrichment analysis showed that VEGFA and IL6 may be involved in the regulation of the PI3K-Akt signaling pathway. Moreover, IL6 was also found to be enriched in cytokine-cytokine receptor interaction signaling pathway. In the ceRNA regulatory network, a large number of miRNAs and lncRNAs were found. In the transcription factor regulatory network, the transcription factor SP1 was correlated with VEGFA, SYP and IL6. It is also predicted that drugs related to key DEmRNAs such as CARBOPLATIN, FENTANYL and CILOSTAZOL may contribute to the treatment of IA. In addition, it was also found that SVM and RF models based on key DEmRNAs may be potential markers for diagnosing IA and unruptured intracranial aneurysm (UIA), respectively. The expression trend of key DEmRNAs verified by real-time PCR was consistent with the bioinformatics analysis results.

**Conclusion:**

The identification of molecules and pathways in this study provides a theoretical basis for understanding the immune related molecular mechanism of IA. Meanwhile, the drug prediction and diagnosis model construction may also be helpful for clinical diagnosis and management.

**Supplementary Information:**

The online version contains supplementary material available at 10.1186/s12863-023-01121-w.

## Introduction

Intracranial aneurysm (IA) is a common cerebrovascular disease. Most of unruptured intracranial aneurysms (UIAs) are incidentally found, asymptomatic and typically benign [1]. The risk of rupture increases with age, aneurysm size, and onset of symptoms [2]. Rupture of an aneurysm can have devastating consequences for the patient. Computerized tomography (CT) and digital subtraction angiography (DSA) are commonly used diagnostic tests [3]. Despite advances in medical technology in recent years, the prognosis for ruptured intracranial aneurysm (RIA) remains poor. Therefore, it is necessary to identify new diagnostic biomarkers to aid the early detection and management of IA.

Previous studies have found that the formation, development and rupture of IA are closely related to immune inflammatory response [4–6]. Loss of balance in CD4 + T cell subsets may contribute to a higher inflammatory state in IA [7]. Th17/Treg was unbalanced in IA, and Th17 cells are positively correlated with the severity of spontaneous subarachnoid hemorrhage (SAH) induced by IA [8]. In addition, interleukin-2 (IL-2) can also significantly enhance the function of Treg cells in IA patients [9]. In addition, IA also exhibits abundant immune cell infiltration and activation of immune-related pathways [10]. The above results suggest that exploring the immune mechanism of IA is beneficial to deepen the understanding of the disease, which in turn can help in treatment and management. However, the immune mechanism of IA is more complicated, and it is unclear so far. Therefore, it is necessary to continue to explore the immune related molecular mechanism of IA.

Machine learning is an emerging field of medicine, and a large number of machine learning algorithms are often used to process medical data and perform feature selection on variables to play an important role in disease detection, diagnosis and treatment [11, 12]. Cytoscape-cytohubba plug-in provides 11 topological analysis methods, which can mine hub genes by ranking nodes in the network according to network characteristics [13]. In this study, machine learning and 6 algorithms in the cytoscape-cytohubba plug-in were used to identify the key immune types and multicentric differentially expressed mRNAs (DEmRNAs) of IA, respectively. Multicentric DEmRNAs related to key immune cells were screened out as key DEmRNAs by Spearman correlation analysis. Subsequently, diagnostic models, competing endogenous RNA (ceRNA) regulatory network and transcription factor regulatory network were also constructed based on key DEmRNAs.

## Materials and methods

### Data sources and processing

Firstly, “intracranial aneurysm” was used as the key word to search in gene expression omnibus (GEO) database [14]. Then, cell line or animal level studies and single-sample studies were excluded. Finally, GSE122897, GSE54083, GSE15629 and GSE75436 datasets were included in this study (Table 1). Among which, GSE122897, GSE54083 and GSE15629 datasets were the discovery cohort, and GSE75436 dataset was the verification cohort. GPL platform annotation file was used to annotate gene expression profile, and gene probe was converted into gene symbol. Multiple probes corresponding to the same gene were averaged. For three datasets in the discovery cohort, batch effects were removed using the combat function in “sva” package.

**Table 1:** Details of 4 datasets included in the study

GEO ID	Samples	Platform	Source	Type
GSE122897	Control: UIA: RIA = 16: 21: 22	GPL16791	tissue	mRNA
GSE54083	Control: UIA: RIA = 10: 5: 8	GPL4133	tissue	mRNA
GSE15629	Control: UIA: RIA = 5: 6: 8	GPL6244	tissue	mRNA
GSE75436	Control: IA = 15: 15	GPL570	tissue	mRNA

*UIA* unruptured intracranial aneurysm, *RIA* ruptured intracranial aneurysm, *IA* intracranial aneurysm

### Identification and functional analysis of DEmRNAs

The “limma” package was used for differential expression analysis to obtain DEmRNAs of IA. The screening criterion for DEmRNAs was set as false discovery rate (FDR) < 0.05, |log_2_ fold change|> 1 (|log_2_ FC|> 1). To understand the function of DEmRNAs, GO and KEGG [15–17] function enrichment analysis was performed based on the David database (https://david.ncifcrf.gov/). FDR < 0.05 was considered significant.

### Identification of key immune cells

The ssGSEA algorithm was used to quantify the relative abundance of each immune cell infiltration in the immune microenvironment (IME) of the IA and control samples. Sets of genes that mark each immune cell type were obtained from Charoentong’s study [18]. The Wilcoxon test was used to statistically analyze the difference of immune cell infiltration between IA and control groups. The correlation between each immune cell in IA and control groups was also analyzed. LASSO regression analysis of the “glmnet” package was performed to identify the first group of candidate key immune cells from 23 types of immune cells. In addition, the randomForest algorithm in the “randomForest” package was used to rank the importance of 23 kinds of immune cells from large to small according to the value of mean decrease accuracy. Then, the top 25% (6) immune cells were selected as the second group of candidate key immune cells. Subsequently, the intersection between candidate key immune cells in the first and second group, and differentially infiltrating immune cells was taken as key immune cells.

### Identification of key DEmRNAs

A protein–protein interaction (PPI) network was constructed based on string database (https://cn.string-db.org/) to study the regulatory relationship between DEmRNAs. The interactions considered by the PPI network include known interactions (from curated databases and experimentally determined), predicted interactions (gene neighborhood, gene fusions and gene co-occurrence) and others (textmining, co-expression and protein homology). Cytoscape software was used to visualize the PPI network. Subsequently, multicentric DEmRNAs were screened using betweenness, degree, edge percolated component (EPC), maximal clique centrality (MCC), maximum neighborhood component (MNC) and stress in the cytoscape-cytoHubba plug-in. DEmRNAs in each algorithm were sorted according to “Score”. The intersection DEmRNAs of the top 30 node DEmRNAs of each algorithm were screened by the “UpSet” package. The Spearman correlation was used to analyze the correlation between multicentric intersection DEmRNAs and key immune cells to explore the synergistic effect of immune cells and DEmRNAs in the occurrence and development of IA. DEmRNAs associated with immune cells were regarded as key DEmRNAs. The screening criterion was *P* < 0.05.

### Construction of ceRNA regulation network

Based on the ENCORI (http://starbase.sysu.edu.cn/index.php) database, miRNAs that regulate key DEmRNAs were searched. Then, differentially expressed miRNAs (DEmiRNAs) of IA were identified based on the GSE66239 dataset (10 control tissue samples and 7 IA tissue samples). The screening criterion was *P* < 0.05 and |log2FC|> 1. Subsequently, DEmiRNAs negatively regulated with key DEmRNAs were selected and their corresponding lncRNAs were searched based on the ENCORI database. Finally, a ceRNA regulation network was constructed.

### Construction of transcription factor regulatory network and drug prediction

Transcription factors related to key DEmRNAs were screened out based on TRRUST database (https://www.grnpedia.org/trrust/) to explore the relationship between transcription factors and key DEmRNAs. In order to provide a new perspective for the diagnosis, treatment and research of IA, drugs related to key DEmRNAs were screened out based on DGIdb database (https://dgidb.org/).

### Construction of diagnostic model

The “random forests”, “rpart” and “e1071” packages were used to construct random forest (RF), decision tree (DT) and support vector machine (SVM) models based on key DEmRNAs, respectively. Receiver operating characteristic (ROC) analysis was performed using the “pROC (version 1.15.3)” package. The accuracy of model was evaluated by area under curve (AUC). Higher value of the AUC indicates the higher diagnostic accuracy [19]. AUC > 0.7 indicates good diagnostic accuracy. Diagnostic accuracy of model was also validated on the GSE75436 dataset. In addition, the diagnostic accuracy of individual key DEmRNAs was also analyzed.

### Real time-PCR validation

Inclusion criteria for patients with IA were confirmed by digital subtraction angiography (DSA) and the patients were Chinese over 18 years of age. Patients with other cerebral hemangiomas, malignancies, severe complications, ongoing pregnancy or lactation, and incomplete clinical information were excluded. The individuals in the control group were gender and age matched with the IA group and had no disease. Those individuals with a family history of IA were ongoing pregnancy or lactation was excluded.

Blood samples from 10 healthy individuals and 8 IA patients were included in this study according to the above screening criteria. Detailed clinical information is shown in Table S1. RNAliquid ultra-speed whole blood (liquid sample) total RNA extraction kit was used to extract total RNA. FastQuant cDNA synthesis kit and SuperReal PreMix Plus (SYBR Green) were used to synthesize cDNA and perform real time-PCR, respectively. GAPDH and ACTB are internal reference genes. Each experiment was repeated three times. 2^−ΔΔCt^ method was used for relative quantitative analysis of data [20]. The present study was approved by the Ethics Committee of The First Affiliated Hospital of Anhui Medical University (B2020003). Written informed consent was obtained from all participants.

### Statistical analysis

All statistical analyses were performed in R software (version 3.5.3). The Wilcoxon test was used to statistically analyze the difference of immune cell infiltration between IA and control groups, as well as the difference of key DEmRNAs expression between UIA, RIA and normal control groups. Spearman correlation was used to analyze the correlation between multicentric intersection DEmRNAs and key immune cells. In real time-PCR, t-test was used to evaluate the statistical significance.

## Results

### Analysis of DEmRNAs

**Fig. 1:**
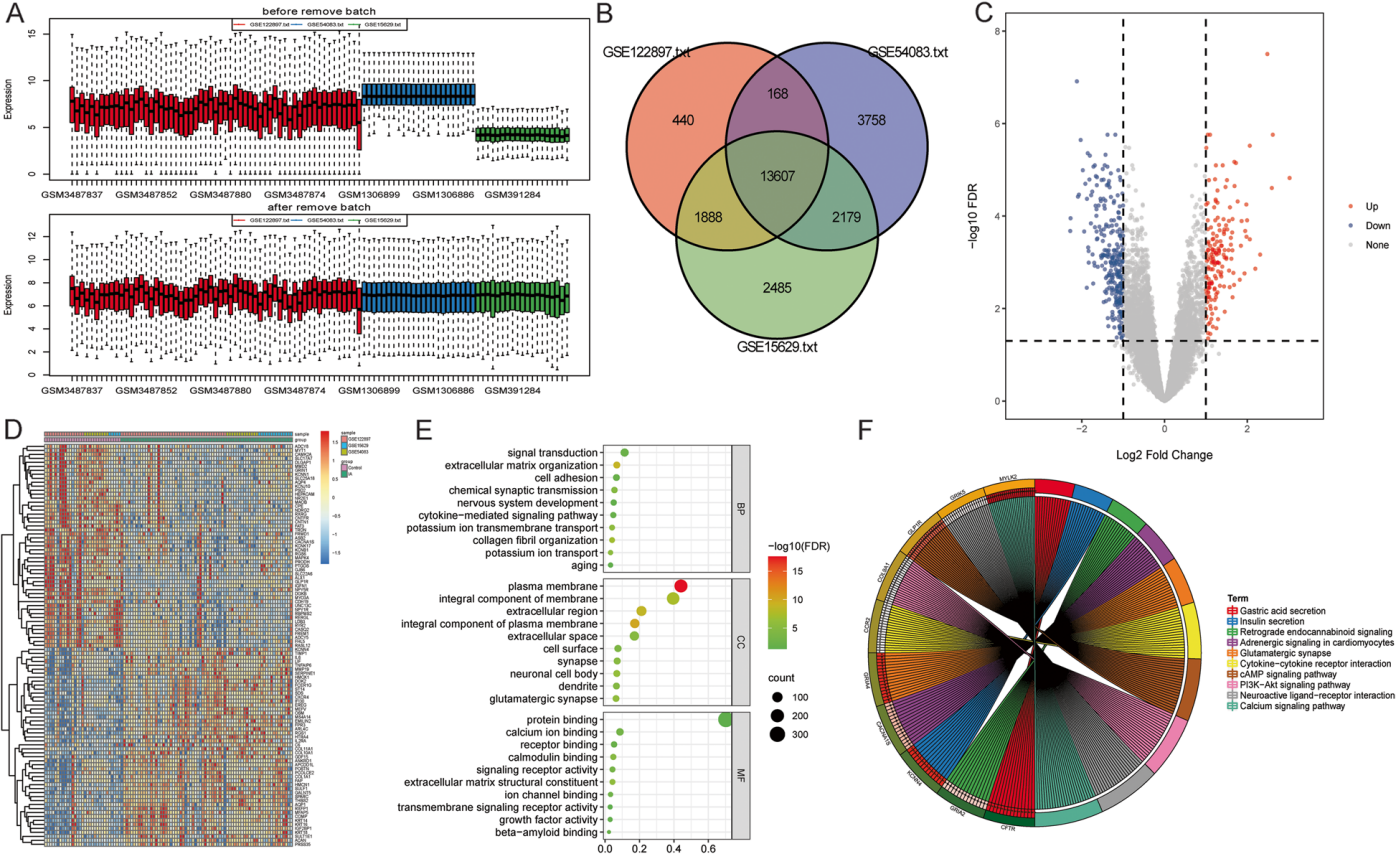
Identification and functional analysis of DEmRNAs. **A**: Boxplot of batch effect of mRNA; **B**: Venn diagram of intersection mRNAs of GSE122897, GSE54083 and GSE15629 datasets; **C**: Volcano map of DEmRNAs; **D**: Heat map of top 50 DEmRNAs; **E**: Bubble plot of top 10 biological process (BP), cellular component (CC) and molecular function (MF) of GO terms; **F**: Circle map of the top 10 signaling pathways of KEGG term

After pretreatment of the original data, a total of 13,607 mRNAs were identified in the discovery cohort (Fig. 1A and B). According to FDR < 0.05 and |log2 FC|> 1, 448 (169 up-regulated and 279 down-regulated) DEmRNAs were identified in IA group. Volcano plot of DEmRNAs and heatmap of the top 50 DEmRNAs are shown in Fig. 1C and D. Subsequently, GO and KEGG functional enrichment analyses were performed to understand the biological function of DEmRNAs (Fig. 1E and F). In biological process (BP) of GO terms, DEmRNAs were mainly involved in signal transduction and extracellular matrix organization. In cellular component of GO terms, DEmRNAs were mainly distributed in plasma membrane and integral component of membrane. In molecular function (MF) of GO terms, the functions of DEmRNAs mainly include protein binding and calcium ion binding. Moreover, KEGG analysis showed that DEmRNAs were enriched in multiple signaling pathways, such as neuroactive ligand-receptor interaction, PI3K-Akt signaling pathway and cytokine-cytokine receptor interaction. GO and KEGG enrichment results indicate the complexity of molecular mechanisms in IA progression.

### Identification of key immune cells in IA

**Fig. 2:**
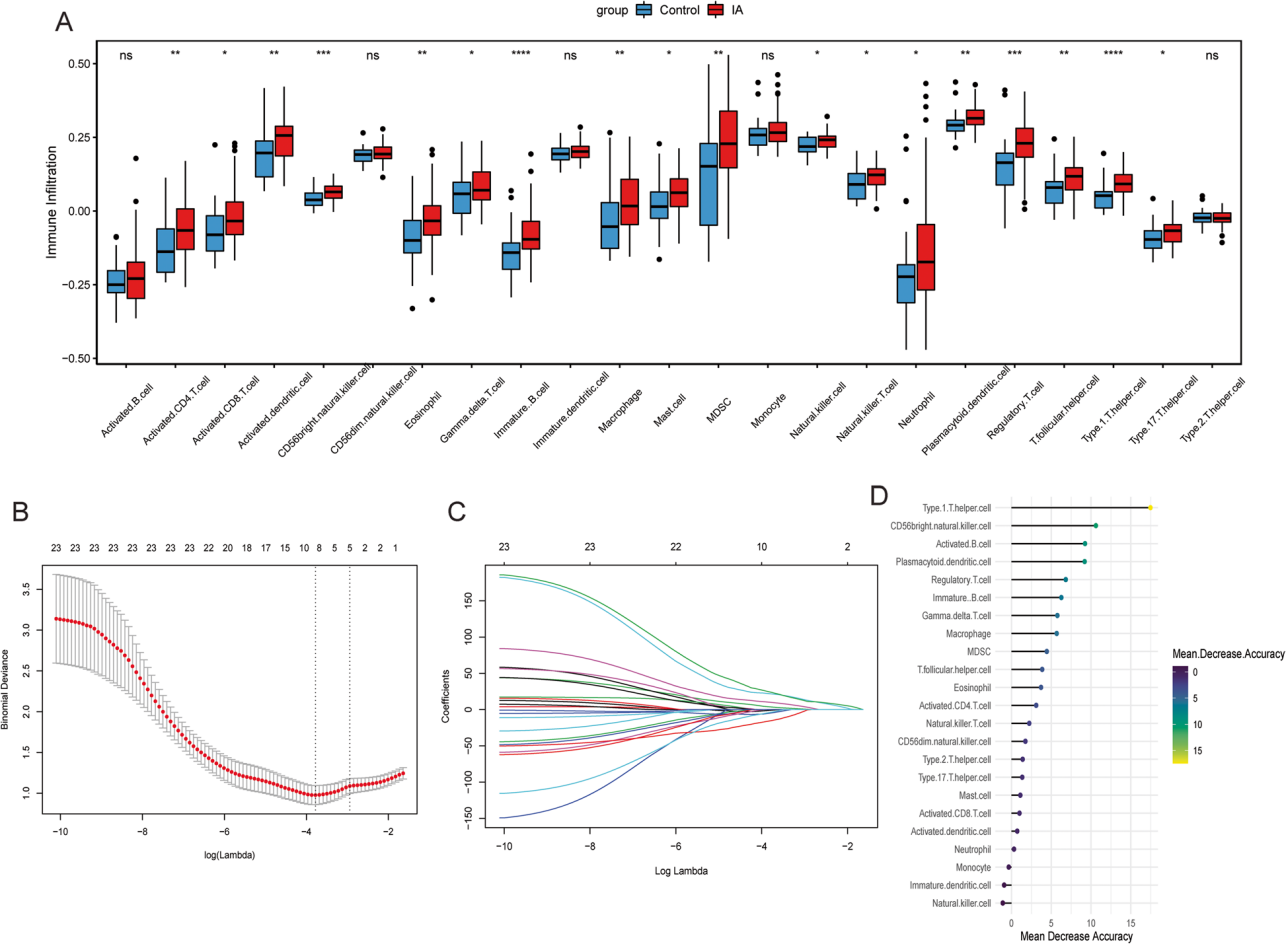
Identification of key immune cells. **A**: Difference analysis of immune cell infiltration between IA group and normal control group. The Wilcoxon test was used to statistically analyze the difference of immune cell infiltration between IA and normal control groups. * represents *P* < 0.05; ** represents *P* < 0.01; *** represents *P* < 0.001; **** represents *P* < 0.0001; ns represent no significant difference. *P* < 0.05 was considered statistically significant. **B**: The partial likelihood deviance for the lasso regression; **C**: The lasso regression analysis; **D**: Mean decrease accuracy ranking of 23 immune cells

The ssGSEA method was used to evaluate infiltration status of 23 immune cells in normal control group and IA group. The results showed that Activated B cell, CD56dim natural killer cell, Immature dendritic cell, Monocyte and Type 2 T helper cell had no significant difference between the two groups, while the infiltration levels of other 18 immune cells in IA group were significantly higher than that in normal control group (Fig. 2A). CD56bright natural killer cell, CD56dim natural killer cell, Immature B cell, Monocyte and Type 1 T helper cell were selected as candidate key immune cells in the first group of by LASSO regression (Fig. 2B and C). Then, CD56bright natural killer cell, Activated B cell, Type 1 T helper cell, Plasmacytoid dendritic cell, Regulatory T cell and Immature B cell were selected as candidate key immune cells in the second group of according to the value of mean decrease accuracy (Fig. 2D). The CD56bright natural killer cell, Immature B cell and Type 1 T helper cell obtained by the intersection of candidate key immune cells in two groups, and differential immune cells were regarded as key immune cells.

### Identification of key DEmRNAs in IA

**Fig. 3:**
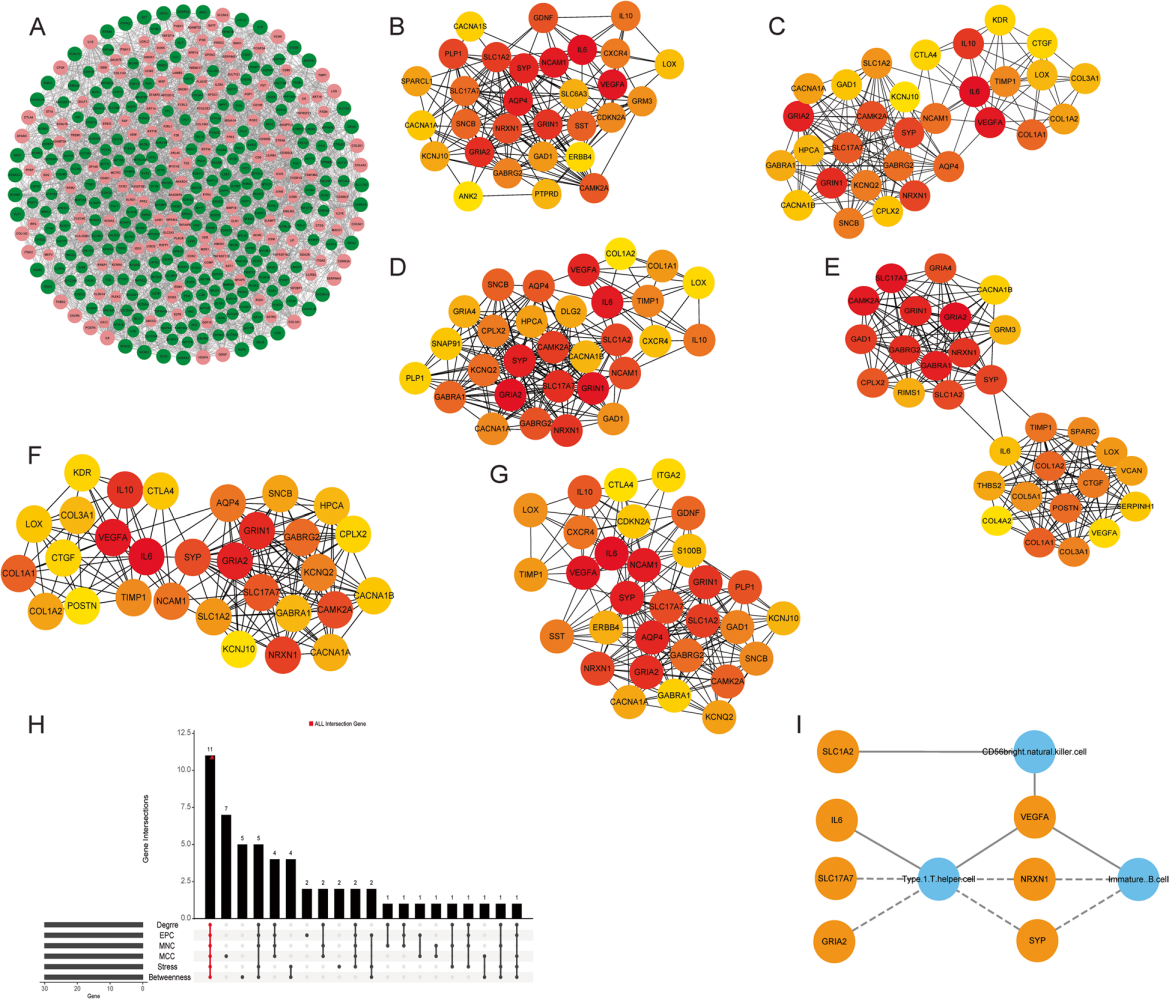
Identification of key DEmRNAs. **A**: PPI network of DEmRNAs. Red and green represent up-regulated and down-regulated DEmRNAs, respectively. **B**: PPI network of the top 30 DEmRNAs scored by the Betweennes algorithm. The color represents the importance of the DEmRNA in the algorithm, the darker the more important. **C**: PPI network of the top 30 DEmRNAs scored by the Betweennes algorithm. The color represents the importance of the DEmRNA in the algorithm, the darker the more important. **D**: PPI network of the top 30 DEmRNAs scored by the Degree algorithm. The color represents the importance of the DEmRNA in the algorithm, the darker the more important. **E**: PPI network of the top 30 DEmRNAs scored by the EPC algorithm. The color represents the importance of the DEmRNA in the algorithm, the darker the more important. **F**: PPI network of the top 30 DEmRNAs scored by the MCC algorithm. The color represents the importance of the DEmRNA in the algorithm, the darker the more important. **G**: PPI network of the top 30 DEmRNAs scored by the Stress algorithm. The color represents the importance of the DEmRNA in the algorithm, the darker the more important. **H**: Screening of multicentric DEmRNAs; **I**: Correlation between key immune cells and key DEmRNAs. Orange represents DEmRNAs, blue represents immune cells, solid line represents positive correlation, and dashed line represents negative correlation

A PPI network of DEmRNAs was constructed based on string database (Fig. 3A). Then, the betweenness, degree, EPC, MCC, MNC and stress algorithms in cytoscape-cytoHubba plug-in were used to screen multicentric DEmRNAs based on PPI network. The top 30 DEmRNAs in each algorithm were screened according to the “Score” (Fig. 3B-G and Table S2). Subsequently, 11 multicentric intersection DEmRNAs (GRIA2, SLC17A7, CAMK2A, GRIN1, GABRG2, NRXN1, SYP, SLC1A2, LOX, IL6 and VEGFA) were screened using the “UpSet” package (Fig. 3H and Table S2). Spearman correlation analysis revealed that only 7 multicentric intersection DEmRNAs were associated with 3 key immune cells (*P* < 0.05) (Fig. 3I).

### Construction of regulatory network and drug prediction

**Fig. 4:**
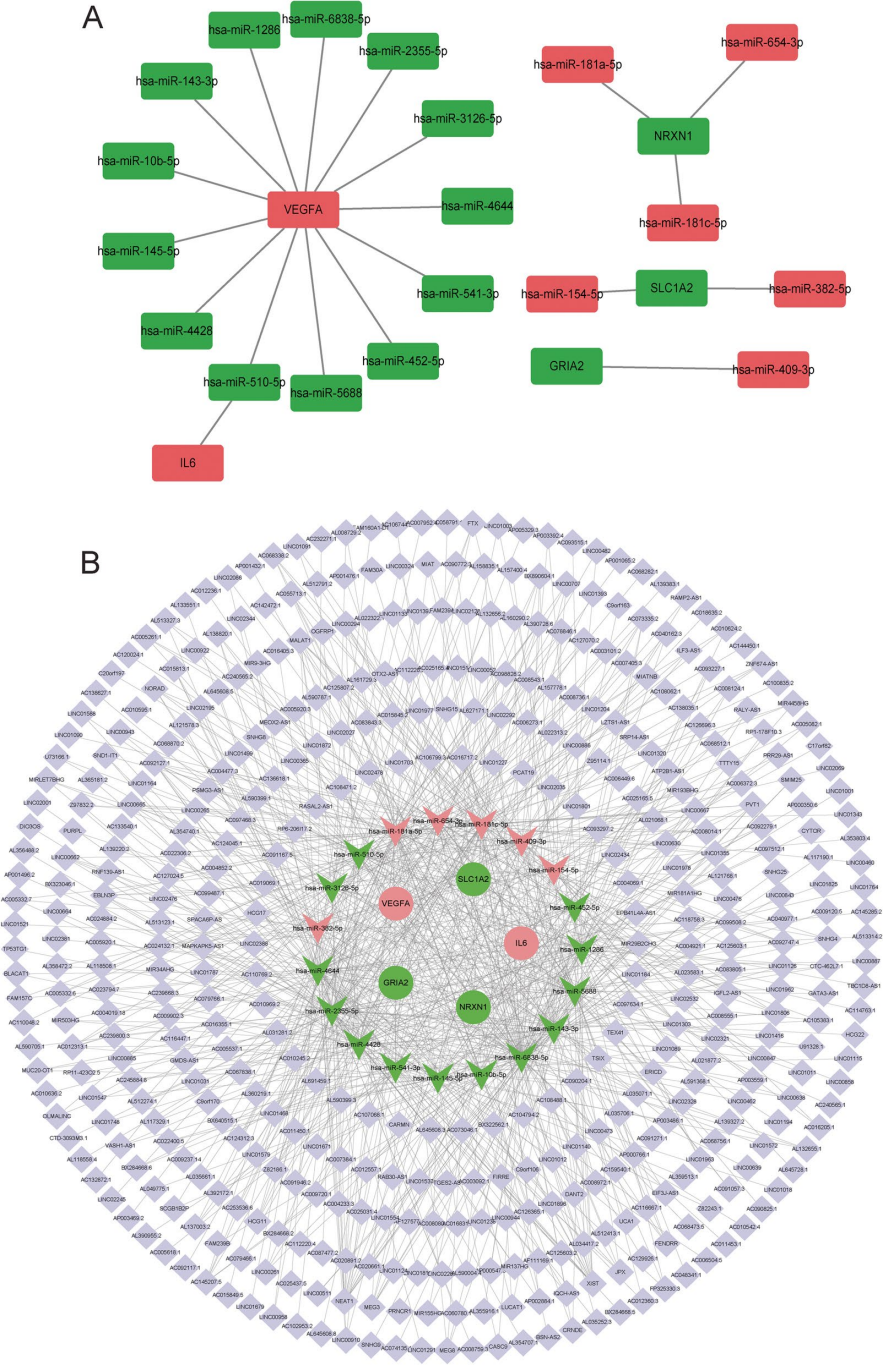
Construction of a ceRNA regulatory network. **A**: DEmRNAs-DEmiRNAs negative regulatory network. Red and green represent up-regulated and down-regulated, respectively. **B**: CeRNA regulatory network. Square, V-shape and circle represents lncRNAs, DEmiRNAs and DEmRNAs, respectively

**Fig. 5:**
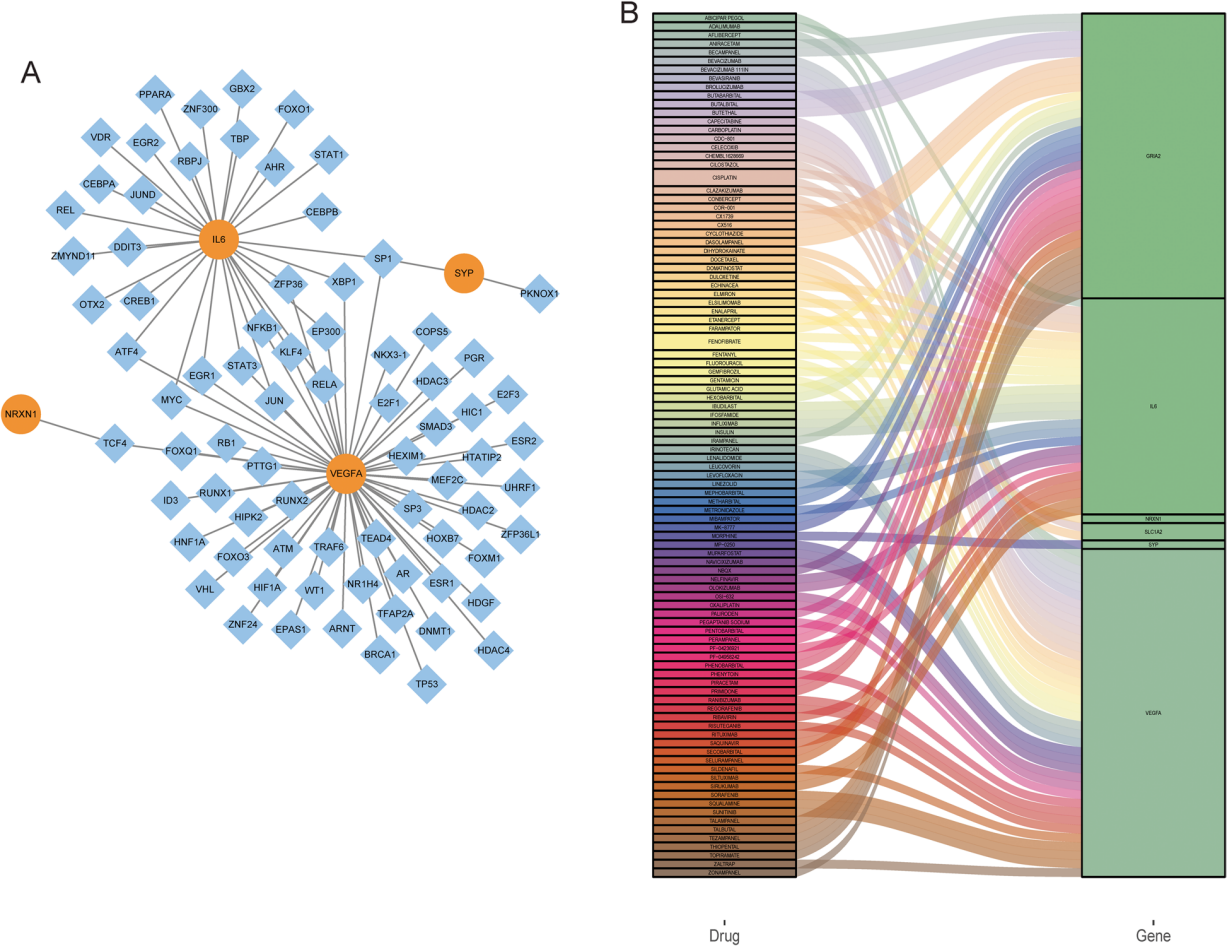
Construction of transcription factor regulatory network **A** and drug prediction **B** of key DEmRNAs

A total of 20 negatively regulated DEmRNAs-DEmiRNAs relationship pairs (including 5 DEmRNAs and 19 DEmiRNAs) were obtained based on ENCORI database and GSE66239 dataset analysis (Fig. 4A). Subsequently, lncRNAs corresponding to 19 DEmiRNAs were found based on the ENCORI database, followed by the construction of the ceRNA network (Fig. 4B). This further indicates the complexity of the molecular mechanism of IA. To understand the correlation between key DEmRNAs and transcription factors, transcription factor regulatory network was constructed (Fig. 5A). In the network, some related transcription factors were identified, such as VEGFA, SYP, NRXN1 and IL6. Moreover, the transcription factor SP1 was correlated with VEGFA, SYP and IL6. In addition, related drugs of key DEmRNAs were also predicted (Fig. 5B). The results showed that there were 33 drugs related to GRIA2, 25 drugs related to IL6, 1 drug related to NRXN1, 2 drugs related to SLC17A7, 1 drug related to SYP, 38 drugs related to VEGFA, and no related drugs to SLC17A7. Identification of these drugs may aid in the treatment and management of IA.

### Diagnostic analysis

**Fig. 6:**
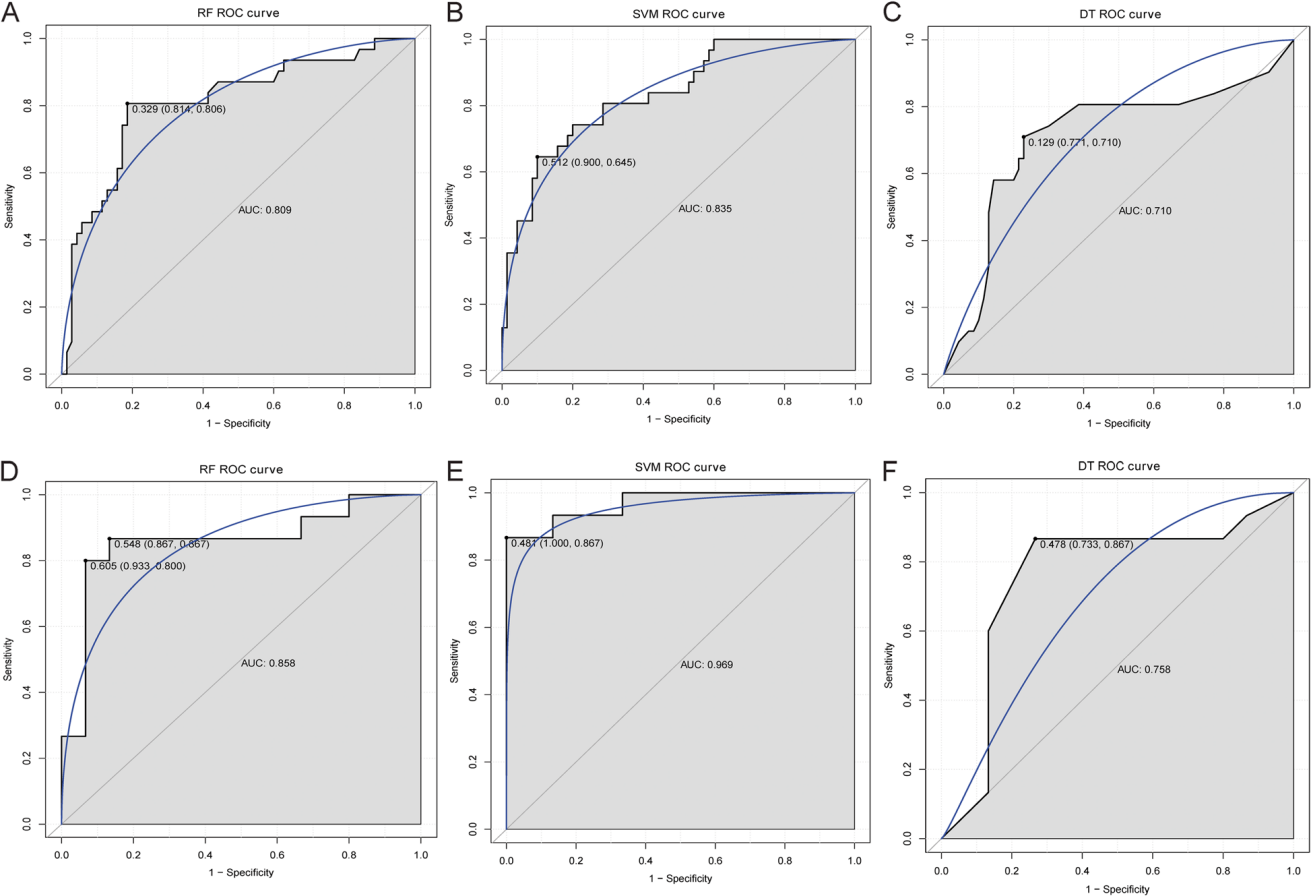
The construction of diagnostic models based on normal control and IA groups. A: Diagnostic accuracy analysis of RF model in discovery cohort; B: Diagnostic accuracy analysis of SVM model in discovery cohort; C: Diagnostic accuracy analysis of DT model in discovery cohort; D: Diagnostic accuracy analysis of RF model in verification cohort; E: Diagnostic accuracy analysis of SVM model in verification cohort; F: Diagnostic accuracy analysis of DT model in verification cohort. ROC, receiver operating characteristic; AUC, area under curve

RF, SVM and DT models all had high diagnostic accuracy in the discovery cohort (AUC > 0.7), and SVM model had the highest diagnostic accuracy (AUC = 0.835) (Fig. 6A-C). Similarly, the RF, SVM and DT models all had high diagnostic accuracy in the verification cohort, and the SVM model had the highest diagnostic accuracy (Fig. 6D-F). Subsequently, ROC analysis of key DEmRNAs showed that AUC values of NRXN1, SLC1A2, SLC17A7, IL6, VEGFA and SYP were all greater than 0.7 (Supplementary Fig. 1). This suggests that NRXN1, SLC1A2, SLC17A7, IL6, VEGFA and SYP may be potential diagnostic biomarkers for IA. Furthermore, the diagnostic accuracy of the SVM model was higher than that of all the individual key DEmRNAs. This further suggests that SVM model may have important significance in clinical diagnosis.

**Fig. 7:**
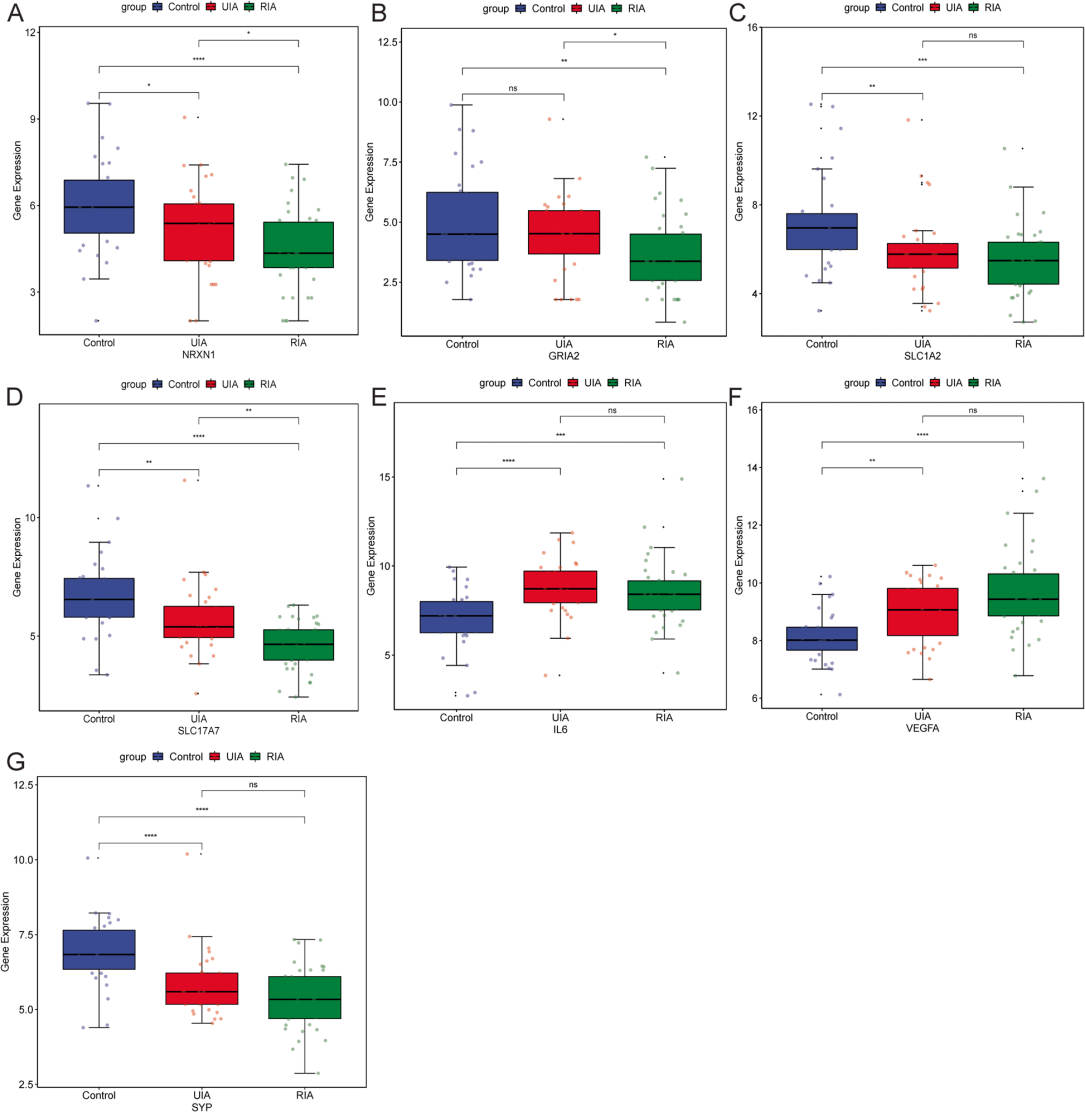
Expression analysis of key DEmRNAs in UIA, RIA and normal control groups. **A**: Expression analysis of NRXN1 in UIA, RIA and normal control groups; **B**: Expression analysis of GRIA2 in UIA, RIA and normal control groups; **C**: Expression analysis of SLC1A2 in UIA, RIA and normal control groups; **D**: Expression analysis of SLC17A7 in UIA, RIA and normal control groups; **E**: Expression analysis of IL6 in UIA, RIA and normal control groups; **F**: Expression analysis of VEGFA in UIA, RIA and normal control groups; **G**: Expression analysis of SYP in UIA, RIA and normal control groups. The Wilcoxon test was used to statistically analyze the expression difference of key DEmRNAs expression between UIA, RIA and normal control groups. * represents *P* < 0.05; ** represents *P* < 0.01; *** represents *P* < 0.001; **** represents *P* < 0.0001; ns represents no significant difference. *P* < 0.05 was considered statistically significant

**Fig. 8:**
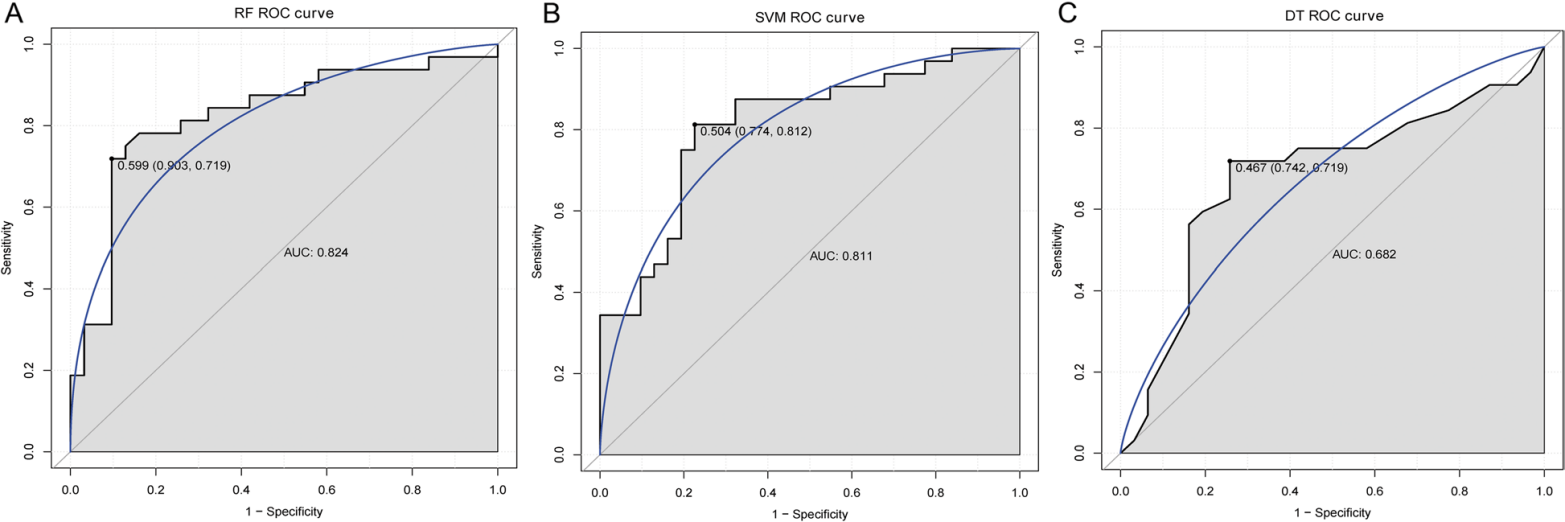
The construction of diagnostic models based on normal control and UIA groups. **A**: Diagnostic accuracy analysis of RF model in discovery cohort; **B**: Diagnostic accuracy analysis of SVM model in discovery cohort; **C**: Diagnostic accuracy analysis of DT model in discovery cohort. ROC, receiver operating characteristic; AUC, area under curve

In addition, IA group was divided into UIA group and RIA group for further study. The Wilcoxon test was used to statistically analyze the difference of key DEmRNAs expression between UIA, RIA and normal control groups. Compared with the normal control group, the expressions of NRXN1, GRIA2, SLC1A2, SLC17A7 and SYP were significantly down-regulated in the RIA group, while the expressions of IL6 and VEGFA were significantly up-regulated (Fig. 7). Compared with the normal control group, the expressions of NRXN1, SLC1A2, SLC17A7 and SYP were remarkably down-regulated in the UIA group, while the expressions of IL6 and VEGFA were remarkably up-regulated. However, there was no significant difference in the expression of GRIA2 between the normal control group and the UIA group (Fig. 7). Subsequently, RF, SVM and DT models were constructed based on key DEmRNAs to distinguish normal control and UIA groups. The result showed that the RF model had the highest diagnostic accuracy (AUC = 0.824) (Fig. 8). Subsequently, ROC analysis of key DEmRNAs showed that AUC values of SLC1A2, SLC17A7, IL6, VEGFA and SYP were all greater than 0.7 (Supplementary Fig. 2). This suggests that SLC1A2, SLC17A7, IL6, VEGFA and SYP may be potential diagnostic biomarkers for UIA. Furthermore, the diagnostic accuracy of the RF model was higher than that of all the individual key DEmRNAs. This further suggests that the RF model may have an important role in clinically distinguishing normal control and UIA groups.

### Expression validation of NRXN1, GRIA2, SLC1A2, SLC17A7, IL6, VEGFA and SYP by real time-PCR

**Fig. 9:**
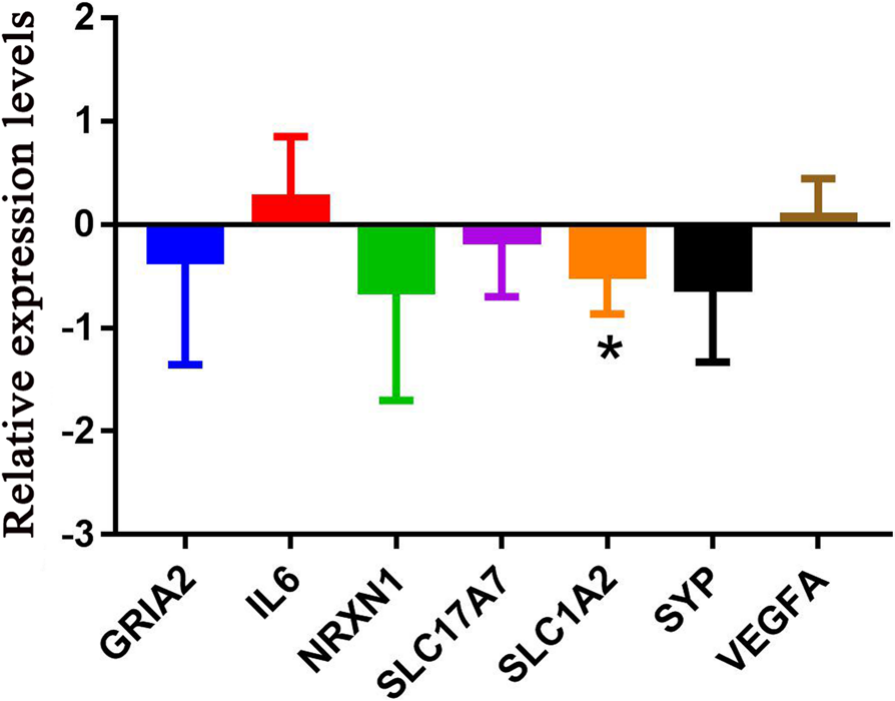
Expression validation of NRXN1, GRIA2, SLC1A2, SLC17A7, IL6, VEGFA and SYP by real time-PCR. * represents *P* < 0.05, *P* < 0.05 was considered statistically significant

Expression validation of NRXN1, GRIA2, SLC1A2, SLC17A7, IL6, VEGFA and SYP was performed by real time-PCR. Primers used for real time-PCR are shown in Table 2. Compared with the normal control group, there was a down-regulated expression trend of NRXN1, GRIA2, SLC1A2, SLC17A7 and SYP in the IA group, while there was an up-regulated trend of IL6 and VEGFA (Fig. 9). Real time-PCR validation results were consistent with the bioinformatics analysis results. However, most of the key DEmRNAs lacked significant differences in real time-PCR expression validation, which may be due to the small sample size. Therefore, a large number of samples are needed to collect for further research.

**Table 2:** Sequences of primers used for real time-PCR verification

Primer name	Primer sequence (5’ to 3’)
GAPDH-F (internal reference)	5-CTGGGCTACACTGAGCACC-3
GAPDH-R (internal reference)	5-AAGTGGTCGTTGAGGGCAATG-3
ACTB-F (internal reference)	5-GATCAAGATCATTGCTCCTCCT-3
ACTB-R (internal reference)	5-TACTCCTGCTTGCTGATCCA-3
GRIA2-F	5-CACATCATTTTGCGGAACACT-3
GRIA2-R	5-AGCACAGCTTGCAGTGTTGAT-3
IL6-F	5-ACTCACCTCTTCAGAACGAATTG-3
IL6-R	5-CCATCTTTGGAAGGTTCAGGTTG-3
NRXN1-F	5-TTCTGCAACGGACAGATCG-3
NRXN1-R	5-CCCAGGGTCATTGCAGAGT-3
SLC17A7-F	5-TACCTGTTCTGGCTGCTCGT-3
SLC17A7-R	5-CAGAAGTTGGCCACGATGAT-3
SLC1A2-F	5-TGTCCACGACCATCATTGCTG-3
SLC1A2-R	5-TTCTTGAGCTTGGGATTGCCT-3
SYP-F	5-TATGGCCACCTACATCTTCCT-3
SYP-R	5-ACAGGGTCTCTCAGCTCCTTG-3
VEGFA-F	5-CTGTCTTGGGTGCATTGGAGC-3
VEGFA-R	5-AGGGTCTCGATTGGATGGCAG-3

## Discussion

IA is a serious clinical disease. Microarray data analysis is a common method to identify abnormal expression molecules of IA. Previous researchers identified a large number of important molecules related to IA based on weighted gene co-expression network analysis (WGCNA), ferroptosis-related ceRNA network analysis and ClusterONE clustering algorithm analysis [21–23]. Although there are many reports on IA research based on microarray data, analysis methods and research directions involved in these studies are relatively simple. In this study, key immune cells and DEmRNAs in IA were identified based on ssGSEA, LASSO regression, cytoscape-cytohubba plug-in and Pearson correlation. In order to understand the underlying molecular mechanism of key DEmRNAs, ceRNA and transcription factor regulatory networks were constructed. In this study, we not only explored the potential molecular mechanism of IA, but also carried out drug prediction and diagnosis model construction. Overall, this study lays a theoretical foundation for further understanding of the complex molecular mechanism of IA. Meanwhile, the drug prediction and diagnosis model construction may also be helpful for clinical diagnosis and management.

To understand the molecular mechanism associated with IA immunity, 7 key DEmRNAs (NRXN1, GRIA2, SLC1A2, SLC17A7, IL6, VEGFA and SYP) associated with key differential immune cell infiltration (CD56bright natural killer cell, Immature B cell and Type 1 T helper cell) were identified. Some researchers found that neurexin 1 (NRXN1) was closely related to neuropsychiatric diseases [24–26]. NRXN1 may also be a new target for antibody–drug conjugate therapy in small cell lung cancer [27]. Moreover, NRXN is also expressed and functions in the vascular system [28]. Glutamate ionotropic receptor AMPA type subunit 2 (GRIA2), associated with arterial restenosis, regulates vascular smooth muscle cell proliferation and migration [29]. GRIA2 mutations are also associated with neurodevelopmental disorders [30]. Solute carrier family 1 member 2 (SLC1A2), also known as EAAT2 or GLT1, is associated with a variety of neuropsychiatric diseases and is an important regulatory molecule of intracranial glioblastoma [31–33]. Abnormality of solute carrier family 17 member 7 (SLC17A7), also known as VGLUT1, seems to be associated with cognitive function in patients with cerebrovascular disease [34]. Synaptophysin (SYP) has also been found to play a role in a variety of diseases such as colorectal cancer [35], frontotemporal dementia syndrome [36] and epithelioid hemangioendothelioma [37]. So far, we have not found relevant studies on NRXN1, GRIA2, SLC1A2, SLC17A7 and SYP in IA. To our knowledge, this is the first study to show that NRXN1, GRIA2, SLC1A2, SLC17A7 and SYP are down-regulated in IA and associated with immune cells in the IME of IA. NRXN1, GRIA2, SLC1A2, SLC17A7 and SYP may play potential regulatory roles in the progression of IA and may be novel molecular biomarkers of IA. Furthermore, the AUC values of SLC17A7 and SYP were greater than 0.7 in the discovery cohort and validation cohort, suggesting that SLC17A7 and SYP may be potential diagnostic markers for IA.

Plasma interleukin 6 (IL6) is an independent prognostic biomarker that can be used to help identify patients at high risk for poor neurological outcomes following RIA [38]. IL-6 is increased in the serum of estrogen-deficient mice and appears to contribute to the rupture of estrogen-deficient cerebral aneurysm in mice by enhancing macrophage infiltration at the circle of Willis [39]. Vascular endothelial growth factor A (VEGFA) can regulate the apoptosis and activity of IA vascular endothelial cells through lncRNA metastasis-associated lung adenocarcinoma transcript 1 (MALAT1)/miR-143/VEGFA signal axis [40]. In this study, IL6 was positively correlated with Type 1 T helper cell, and VEGFA was positively correlated with CD56bright natural killer cell, Immature B cell and Type 1 T helper cell, suggesting that IL6 and VEGFA may play a role in the immunomodulatory process of IA. In addition, the AUC values of IL6 and VEGFA were greater than 0.7 in the discovery cohort and validation cohort, suggesting that IL6 and VEGFA may be potential diagnostic markers for IA. KEGG functional enrichment analysis showed that VEGFA and IL6 may be involved in the regulation of the PI3K-Akt signaling pathway. One study showed that silencing serine-arginine protein kinase 1 (SPRK1) could inhibit the PI3K/Akt signaling pathway, thereby increasing cell proliferation and vascular remodeling, and reducing the apoptosis rate of vascular smooth muscle cells (VSMCs) in IA rats [41]. Furthermore, T helper (Th) 17/Treg balance in IA requires maintenance of the PI3k/Akt/NF-κB signaling pathway [42]. In addition, IL6 was found to be enriched in cytokine-cytokine receptor interaction signaling pathway. A previous study suggested that cytokine-cytokine receptor interaction signaling pathway may be associated with subarachnoid hemorrhage caused by RIA [43]. Therefore, it is speculated that VEGFA and IL6 may play a role in the formation and development of IA by regulating PI3K-Akt and cytokine-cytokine receptor interaction signaling pathways, which provide a direction for further research on the molecular mechanism of IA.

To further understand the molecular regulatory mechanisms of key DEmRNAs, ceRNA and transcription factor regulatory networks were constructed. In the ceRNA regulatory network, 19 DEmiRNAs were included. Hsa-miR-409-3p can regulate the proliferation and apoptosis of human brain vascular smooth muscle cells [44]. Hsa-miR-143-3p is reduced in patients with aneurysmal subarachnoid hemorrhage and is associated with poor prognosis, and hsa-miR-145-5p level is also significantly reduced [45]. These DEmiRNAs were negatively correlated with key DEmRNAs, so we speculated that the role of key DEmRNAs in IA might be regulated by DEmiRNAs. In addition, ceRNA also contains a large number of lncRNAs, and the specific molecular mechanism needs to be further studied. Related transcription factors of VEGFA, SYP, NRXN1 and IL6 were found in the transcription factor regulatory network. The Sp1 transcription factor (SP1) was correlated with VEGFA, SYP and IL6. A study found that SP1 transcriptionally activates pituitary tumor-transforming gene 1 (PTTG1) to regulate the migration and phenotypic transformation of Human aortic vascular smooth muscle cells in AD through MAPK signaling [46]. Moreover, the abnormal expression of SP1 is also associated with the recurrence of meningioma [47]. Therefore, the underlying mechanism of SP1 involvement in IA is worthy of further investigation. In order to provide a new perspective for the diagnosis, treatment and research of IA, drugs related to key DEmRNAs were also screened out based on DGIdb database. CARBOPLATIN can treat metastatic myxomatous cerebral aneurysms [48]. FENTANYL can relieve headache with little side effects after neck clipping of ruptured IA [49]. CILOSTAZOL can effectively prevent cerebral vasospasm and improve prognosis in patients with aneurysmal subarachnoid hemorrhage [50]. In addition, most of the drugs have not been found to be related to the treatment of IA, and their potential value can be explored in future studies.

In this study, RF, SVM and DT models were constructed based on machine learning. For the normal control and IA groups, the SVM model had the highest diagnostic accuracy in the discovery and validation cohorts. Furthermore, the diagnostic accuracy of the SVM model was higher than that of all the individual key DEmRNAs. Therefore, it is speculated that SVM model is of great significance in the clinical diagnosis of control and IA. UIA is mostly asymptomatic and difficult to diagnose. RF, SVM and DT models were constructed based on key DEmRNAs to distinguish normal control and UIA groups. The result showed that the RF model had the highest diagnostic accuracy (AUC = 0.824). This implies that the RF model may have an important role in clinically distinguishing the normal control and UIA groups. Both the SVM model and the RF model considered as potential diagnostic tools are based on public datasets, and a large number of clinical samples are needed to collect for further research if they can be used clinically.

This study has some limitations. Firstly, the sample size in the RT-PCR is small, and a large number of samples are needed to collect for further research. Secondly, the diagnostic model obtained in this experiment needs to be verified in a large number of clinical samples. Finally, the specific mechanisms of the identified molecules require extensive in vitro studies. In a word, the identification of molecules and pathways in this study provides a theoretical basis for understanding the immune related molecular mechanism of IA.

## Supplementary Information

**Additional file 1:** **Table S1.** Clinicalinformation of IA patients and normal controls in the real time-PCR

**Additional file 2:** **Table S2.** screening ofmulticentric DEmRNAs

**Additional file 3:** **Supplementary Figure 1.** Diagnostic accuracy analysis of NRXN1 (A), GRIA2 (B), SLC1A2 (C), SLC17A7 (D),IL6 (E), VEGFA (F) and SYP (G) based on control group and IA group.ROC, receiver operatingcharacteristic; AUC, area under curve

**Additional file 4:** **Supplementary Figure 2.** Diagnostic accuracy analysis of NRXN1 (A), GRIA2 (B), SLC1A2 (C), SLC17A7 (D),IL6 (E), VEGFA (F) and SYP (G) based on control group and UIA group.ROC, receiver operatingcharacteristic; AUC, area under curve

## Data Availability

All data generated or analyzed during this study are included in this published article. We searched for IA public gene expression data from GEO (http://www.ncbi.nlm.nih.gov/geo) databases. The accession numbers are GEO: GSE122897, GEO: GSE54083, GEO: GSE15629 and GEO: GSE75436, respectively.
